# Preparation and Characterization of MUC-30-Loaded Polymeric Micelles against MCF-7 Cell Lines Using Molecular Docking Methods and In Vitro Study

**DOI:** 10.1155/2021/5597681

**Published:** 2021-05-28

**Authors:** Norased Nasongkla, Patoomratana Tuchinda, Bamroong Munyoo, Komgrit Eawsakul

**Affiliations:** ^1^Department of Biomedical Engineering, Faculty of Engineering, Mahidol University, Nakhon Pathom 73170, Thailand; ^2^Department of Chemistry and Center of Excellence for Innovation in Chemistry, Faculty of Science, Mahidol University, Nakhon Bangkok 10400, Thailand; ^3^Excellence Center for Drug Discovery (ECDD), Faculty of Science, Mahidol University, Bangkok 10400, Thailand; ^4^Department of Chemistry, Faculty of Science, Mahidol University, Bangkok 10400, Thailand; ^5^School of Medicine, Walailak University, Nakhon Si Thammarat 80160, Thailand; ^6^School of Allied Health Sciences and Research Excellence Center for Innovation and Health Products (RECIHP), Walailak University, Nakhon Si Thammarat 80160, Thailand

## Abstract

MUC-30 is a hydrophobic compound which is active against the MCF-7 cancer cell line. In this study, MUC-30 was loaded in polymeric micelles to improve the water solubility and release rate. For prolonged MUC-30 release, MUC-30 was encapsulated in polymeric micelles using PEG-*b*-PLA and PEG-*b*-PCL as materials. Micelles prepared with 1 : 9 *w* per *w* ratios by film hydration achieved the highest entrapment efficiency (EE%). The EE% of MUC-30-loaded PEG-*b*-PCL micelles was approximately 30% greater than that of PEG-*b*-PLA micelles, due to the different H-bond formations between MUC-30 and the polymer membrane (PCL, 4; PLA, 3). The cytotoxic activity of MUC-30 against EGFR theoretically presented 399.31 nM (IC_50_ = 282.26 ng/mL) by molecular docking. In vitro cytotoxic activity of MUC-30 was confirmed by MTT assay. MUC-30 (IC_50_ = 11 ± 0.39 ng/mL) showed three-fold higher activity over MUC-30-loaded PEG-*b*-PLA micelles (IC_50_ = 37 ± 1.18 ng/mL) and two-fold higher over PEG-*b*-PCL micelles (IC_50_ = 75 ± 3.97 ng/mL). This was due to the higher release rate of MUC-30 from PEG-*b*-PLA micelles compared to PEG-*b*-PCL micelles. Therefore, MUC-30-loaded PEG-*b*-PLA micelles could be a promising candidate for breast cancer chemotherapy.

## 1. Introduction

Worldwide, in 2018, the most common cancer in women was breast cancer, with approximately 2.1 million cases [1]. Most breast cancer deaths are due to migration of the tumor to other parts of the body and the complexity of molecular mechanism. The effectiveness of MUC-30 lies in its ability to bind with targeted proteins to overcome these limitations involving in the development and growth of breast cancer caused from drug-resistant mechanisms [2]. Breast cancer usually presents the following proteins: estrogen receptor (ER) [3], progesterone receptor (PR) [4], epidermal growth factor receptor (EGFR) [5], and human epidermal growth factor receptor 2 (HER2) [6]. In addition, proteins that are related to drug resistance are P-glycoprotein (Pgp) [7] and NF-*κ*B activation [8]. Therefore, diminishing the expression of ER, PR, EGFR, HER2, Pgp, and NF-*κ*B should be an important strategy to inhibit the growth and drug resistance of breast cancer cells. To determine protein inhibition, binding affinity of this compound to the mentioned proteins will be evaluated and compared to standard treatment such as tamoxifen [9].

**Figure 1:**
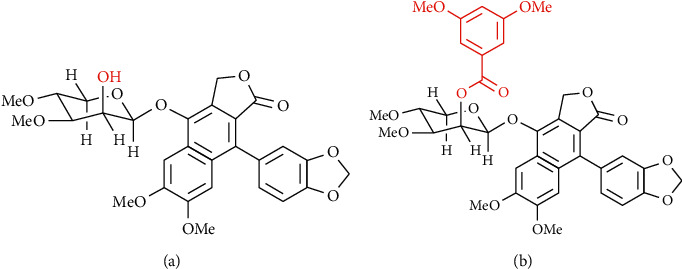
Structure of (a) cleistanthin A and (b) MUC-30.

MUC-30 (Figure 1), a semisynthetic analog of cleistanthin A from *Phyllanthus taxodiifolius* Beille, can be utilized to inhibit breast cancer [10]. Nevertheless, the use of MUC-30 has limitations, i.e., poor water solubility and multidrug resistance (MDR) caused to induce expression of P-glycoprotein (Pgp) and NF-*κ*B activation. To overcome these obstacles, a polymeric micelle from block copolymers was employed to encapsulate MUC-30 within the core. Encapsulation of drugs in these block copolymeric micelles including poly(ethylene glycol)-b-poly (D, L-lactide) (PEG-*b*-PLA) and poly(ethylene glycol)‐b‐poly (*ε*‐caprolactone) (PEG-*b*-PCL) [11] has been proved to increase water solubility of drugs [12] and prevent the development of drug resistance inhibiting ABC-transporter-mediated drug efflux [13–15]. These micelles were proved to be safe in animals [16].

Therefore, in this work, we evaluate MUC-30-loaded polymeric micelles' properties associated with water solubility, drug entrapment, drug release, and the ability of MUC-30 to inhibit MCF-7. Moreover, targeted proteins relating to breast cancer such as ER*α*, PR, EGFR, HER2, Pgp, and NF-*κ*B were analyzed for impact after being treated with MUC-30 by the estimation of IC_50_ values calculated by AutoDock [17]. Results were compared to IC_50_ obtained by MTT assay [18–20].

## 2. Materials and Methods

### 2.1. Materials

#### 2.1.1. Chemical Reagents

Two types of block copolymers, PEG (5 kDa)-b-PCL (5 kDa) and PEG-(5 kDa)-b-PLA (5 kDa), were kindly provided by NanoPolyPEG Co., Ltd. (Thailand). All the organic solvents used in this study were purchased from RCI Lab-Scan Ltd. PBS at pH 7.4 contains potassium chloride (KCl), sodium hydrogenphosphate (Na_2_HPO_4_), sodium chloride (NaCl), disodium, and potassium phosphate monobasic (KH_2_PO_4_). MUC-30 was kindly provided by Prof. Dr. Patoomratana Tuchinda. The MUC-30 compound was purified by HPLC at approximately 99% purity.

#### 2.1.2. Cell Line

A human breast adenocarcinoma cell line (MCF-7) was purchased from the American Type Culture Collection to be used in the cytotoxicity test. It was cultured by the DMEM (Dulbecco's Modified Eagle's medium), which was obtained from Gibco (Grand Island, New York). Most cancer cell lines with the DMEM were able to obtain better growth than the Minimum Essential Medium (MEM) due to the DMEM having four times the number of vitamins and amino acids and 2-fold of glucose. The supplemental agents 10% fetal bovine and 1% penicillin/streptomycin (pen/strep) were purchased from JR Scientific Inc. (Woodland, California) and added to the DMEM. The MCF-7 cell line was cultured in an incubator with a human-like environment at 5% CO_2_ in humidified atmosphere at 37°C.

#### 2.1.3. Water Solubility

The solubility of MUC-30 can also be predicted computationally using the mathematical software COSMOquick. The COSMOquick approach uses a QSPR technique [21] to estimate solubility. In this study, Δ*G*_fus_ has been calculated according to the following equation:

(1) $$\begin{array}{c} \Delta G_{\text{fus}}=\frac{\Delta H_{\text{fus}}-\Delta H_{\text{fus}}\left(1-T\right)}{T_{m}}, \end{array}$$

where Δ*H*_fus_ is the enthalpy of fusion, *T* is set at room temperature, and *T*_*m*_ is the melting temperature for MUC-30. These values were estimated efficiently using COSMOquick.

#### 2.1.4. Molecular Modeling

The targeted protein structure of ER*α* (PDB code: 3ERT), PR (PDB code: 4OAR), EGFR (PDB code: 2J6M), HER2 (PDB code: 3WSQ), Pgp (PDB code: 6QEX), and NF-*κ*B (PDB code: 1SVC) was collected from the Protein Data Bank. The structure of the MUC-30 ligand is as given in Figure 1 which was drawn in ChemSketch 3.5; then, MUC-30 was submitted to the energy minimization tool using Arguslab software [22]. The geometry of MUC-30 was optimized using the semiempirical (PM3) Hamiltonian with Restricted Hartree–Fock (RHF). Both the ligand and targeted proteins were prepared in a PDB format prior to docking using Avogadro software [23].

#### 2.1.5. Building Polymer Surface

Monte-Carlo and molecular dynamics methods were utilized for constructing polymers with surfaces. The polymer structure was optimized using energy constraints. The polymer surface [24] was prepared following a confined surface of PLA at a density of 1.27 g·cm^−3^ and PCL at a density of 1.15 g·cm^−3^. PLA and PCL with twenty-five repeating units were reconstructed in an orthorhombic cell of dimension 36 Å × 36 Å × 18.8 Å and 36 Å × 36 Å × 18.2 Å, respectively.

#### 2.1.6. Docking Simulation

In AutoDock, to encompass the entire ER-binding pocket, the searching grid box with xyz points was set to a size of 82 × 94 × 90, with the grid position at 22.807, 4.785, and 22.682 and the spacing at 0.603 Å. The PR-binding pocket with *xyz* points of size was set at 98 × 106 × 68, with a grid position of 7.586, 31.792, and 12.134 and a spacing of 0.603 Å. The EGFR-binding pocket with *xyz* points of size was set at 126 × 126 × 118, with a grid position at −52.193, −5.861, and −22.264 and a spacing of 0.525 Å. The HER2-binding pocket with *xyz* points of size was set at 118 × 70 × 80, with a grid position at 161.798, 0.581, and 56.51 and a spacing of 0.867 Å. The Pgp-binding pocket with *xyz* points of size was set at 66 × 90 × 126, with a grid position at 173.709, 166.734, and 195.198 and a spacing of 1.0 Å. The NF-*κ*B binding pocket with *xyz* points of size was set at 112 × 126 × 126, with a grid position at 40.522, 14.118, and 28.593 and a spacing for targeted proteins of 0.603 Å. The PLA surface with xyz points of size was set at 110 × 96 × 50, with a grid center located at 17.481, 12.081, and 8.975 and a spacing of 0.5 Å. The PCL surface with *xyz* points of size was set at 126 × 126 × 76, with a grid center at 14.576, 11.985, and10.819 and a spacing of 0.375 Å. The possible docking conformations of the MUC-30 ligand in the targeted proteins and polymer surface were obtained using the Lamarckian genetic algorithm (LGA) with the number of GA runs set to 50. Default settings were used for all other parameters. The results are reported in terms of binding energy (kcal mol^−1^) and inhibition constant (*M*).

#### 2.1.7. Binding Site Analysis

After docking, the docked complexes were visualized in the Discovery Studio to investigate MUC-30 interactions with targeted proteins and the polymer surface [25, 26]. The results were presented in two types: (1) the binding site of the targeted protein for docking is specified. (2) The MUC-30 ligand was docked to the specified prepared polymer surface which is the PLA and PCL surface.

#### 2.1.8. Preparation of MUC-30-Encapsulated Polymeric Micelles

MUC-30-loaded polymeric micelles were fabricated by the film sonication method [12, 27]. MUC-30 and the polymer were dissolved in tetrahydrofuran (THF). To obtain the film, the solvent dissolving mixture was evaporated by using a rotary vacuum evaporator (IK, RV10). After that, distilled water was added to film and subsequently sonicated for 1 min by using Sonic-VibraCell™ (model CV.18, 130 W, 20 kHz).

#### 2.1.9. Particle Size Determination

MUC-30-loaded polymeric micelles were prepared through film sonication; then, the size and size distribution of total MUC-30 entrapped in the polymeric micelles at the concentration of 2 mg·mL^−1^ were determined by laser light scattering (Zetasizer Nano ZS, Malvern).

#### 2.1.10. Water Solubility of MUC-30

UV-Vis spectroscopy was utilized to determine the solubility of the compounds. Firstly, 2.5 mL of THF was used to dissolve 1 mg of MUC-30. The dissolved solution was dropped into 100 mL of water and stirred for 72 h at room temperature to allow THF evaporated [27]. The insoluble drug was removed by refrigerated centrifugation (4°C) for 10 min at 3000 rpm and 0.45 *μ*m syringe filtration. The solution was then lyophilized. The water solubility of MUC-30 was calculated from the total volume of water added after being dissolved in 5 mL of DMSO.

#### 2.1.11. Drug Loading Study

The amount of MUC-30 encapsulated in polymeric micelles was determined by UV-Vis spectroscopy. The freshly prepared micelle solution (10 mL) was purified by refrigerated centrifugation for 10 minutes at 3000 rpm and filtration through a 0.45 *μ*m syringe filter to remove polymer aggregation. After purifying, there are still some unencapsulated drugs. Therefore, a centrifugal filter with a 50 kDa molecular weight cutoff (Millipore, USA), which could separate the drug-loaded micelles located above while unencapsulated drugs fall, was employed to remove unencapsulated MUC-30. The unencapsulated MUC-30 was collected and lyophilized. Lyophilized particles were subsequently measured for the number of MUC-30 by dissolving in chloroform. The lyophilized micelles were also measured by dissolving in chloroform. The absorbance of MUC-30 was recorded at 263 nm. The drug properties such as drug loading density, drug loading efficiency, and yield were calculated using the following equations:

(2) $$\begin{array}{c} \%\text{ drug loading density}=\frac{\text{the amount of drug in micelles}}{\text{the amount of micelles}-\text{free drug}}\times 100,\\ \text{\% drug loading efficiency}=\frac{\text{the amount of drug in micelles}}{\text{the initial amount of drug in system}}\times 100,\\ \text{\% yield}=\frac{\text{total micelle amount of drug}-\text{free drug}}{\text{theoretical total amount of micelle}}\times 100. \end{array}$$

#### 2.1.12. Drug Release Profile

Drug-loaded polymeric micelles were transferred into a dialysis bag with a 50 kDa molecular weight cutoff. The dialysis bag containing drug-loaded polymeric micelle was surrounded by 20 mL of phosphate buffer saline (PBS) at pH 7.4. The release studies were performed at 37°C. At a predetermined time, PBS was taken to measure the amount of MUC-30 at selected time intervals and 20 mL of fresh PBS was replaced [28]. The amount of MUC-30 in a cosolvent of PBS and ethanol was detected using a microplate reader at the excitation wavelength at 260 nm and the emission wavelength at 418 nm. To explain the drug dissolution process, the drug release data were computed using DDsolver [29].

#### 2.1.13. In Vitro Cytotoxicity Test

The in vitro cytotoxicity test was carried out in the Laboratory for Biocompatibility Testing of Medical Devices, Mahidol University. MCF-7 was evaluated by MTT assay. A density of MCF-7 at 5 × 10^3^ cells per 100 *μ*L medium was seeded into 96-well plates. After 1-day incubation, cells were washed once with the medium and the various MUC-30 concentrations prepared in the medium were added. The cell viability was evaluated 72 h after the treatment by MTT assay. Cell survival was defined by a change in the cell color to blue-purple and was conducted by dissolving formazan in dimethyl sulfoxide (DMSO). The color intensity was recorded at 570 nm using a microplate reader (TECAN).

#### 2.1.14. Statistical Analysis

All experiments were carried out in triplicate and are presented as the mean ± standard deviation (SD). An analysis of the data (ANOVA) was conducted using SPSS Statistics 17.0. A *p* value which was less than 0.05 was considered to be statistically significant.

## 3. Results and Discussion

### 3.1. Water Solubility

**Figure 2:**
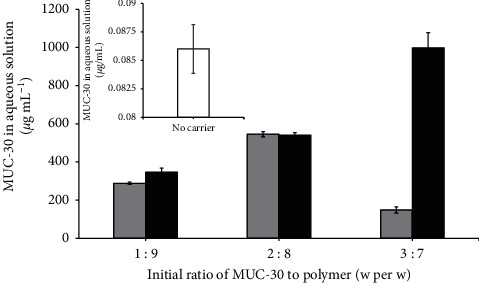
Solubility of MUC30 (white) and MUC30-loaded polymeric micelles at different drug-to-polymer ratios of PEG-*b*-PCL (black) and PEG-*b*-PLA (grey).

This experiment showed that a high ratio between MUC-30 and the PEG-*b*-PCL copolymer increased water solubility, as shown in Figure 2. The ratio at 1 : 9, 2 : 8, and 3 : 7 of MUC-30-loaded PEG-*b*-PCL exhibits an increase in water solubility by 3,000-, 6,000-, and 10,000-fold, respectively, compared to QSPR computational and experimental MUC-30 following 5.44 × 10^−9^ g·L^−1^ and 0.086 *μ*g·mL^−1^, respectively. In contrast, the maximum ratio of MUC-30-loaded PEG-*b*-PLA was 2 : 8, which exhibited an increase in water solubility by 6,000-fold compared to unencapsulated MUC-30. This is due to aggregation of MUC-30-loaded PEG-*b*-PLA at a ratio of 3 : 7.

### 3.2. Size and Drug Loading Content of MUC-30 in Micelles

All ratios of MUC-30-loaded polymeric micelles provide a proper size of micelles (10–200 nm). Data show that the amount of MUC-30 loaded did not affect the size, as shown in Table 1. The large amount of MUC-30 loaded in PEG-*b*-PCL micelles provides smaller particle size than that of MUC-30-loaded PEG-*b*-PLA micelles. This was due to poor water-soluble property of the structure, leading to greater swelling of MUC-30-loaded PEG-*b*-PLA particles. Therefore, particle size was mainly affected by the type of copolymer. The PCL structure encapsulated larger amount of MUC-30 than PLA. MUC-30 and the polymer were selected for further experiments including the release profile and cytotoxicity because this ratio provides the smallest size and high drug loading including drug loading density, encapsulation efficacy, and yield [30]. This study suggested that the ratio at 1 : 9 *w* per *w* of MUC-30 and polymer is the proper proportion to encapsulate MUC-30.

### 3.3. In Vitro Release Study

**Figure 3:**
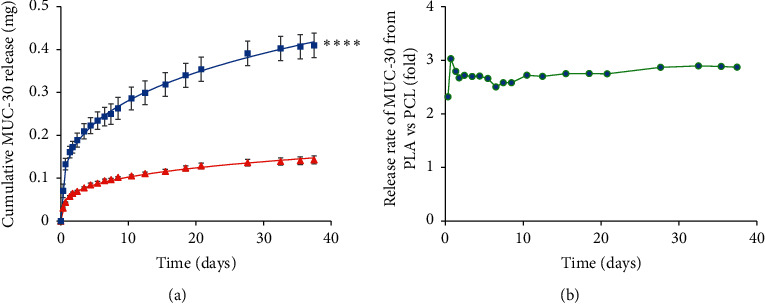
Cumulative release of MUC-30 in mg (a) and fold release rate (b) of MUC-30 from PEG-*b*-PLA (blue square) compared to PEG-*b*-PCL (red triangle) micelles. Experimental data were fitted to the Korsmeyer–Peppas model (solid lines). The symbol *∗∗∗∗* indicates significant statistical difference of MUC-30 release from polymers (*∗∗∗∗*, *P* < 0.0001).

The in vitro release study in PBS at pH 7.4 and 37°C shows that MUC-30 released slower as a result of the PCL structure than the PLA structure and also caused by higher hydrophobicity of PCL compared to PLA, as shown in Figure 3. This is similar to other studies that hydrophobic compounds had a slower release rate than hydrophilic compounds [27, 31].

Eight release models including Baker–Lonsale, first order, Hopfenberg, Hixson–Crowell, Higuchi, Korsmeyer-Peppas, quadratic, and zero order were fitted to find the best-fitted release model and explain the mechanism of drug release. The best-fitting model was the Korsmeyer–Peppas model with *R*^2^ > 0.99 (Tables 2 and 3), with the release of MUC-30 from micelles explained via the following equation:

(3) $$\begin{array}{c} \log\left(\frac{Mt}{M\alpha }\right)=n\;\log\;t+\log\;k, \end{array}$$

where *Mt*/*Mα* is the fraction of cumulative drug release at a specified time, *k* is the constant of drug release rate, *n* is employed to explain the release mechanisms such as diffusion or polymer relaxation and combination mechanisms between diffusion and erosion control. *n* values less than 0.43 are considered to be diffusion, while *n* values between 0.43 and 0.85 indicate polymer relaxation. The *n* values of both MUC-30-loaded polymeric micelles were 0.275 and 0.301, which is less than 0.43, indicating release through diffusion. The *k* value of PCL (2.753) is less than that of PLA (7.236) causing a 2.72-fold slower release of MUC-30 from PCL compared to PLA.

### 3.4. Computational Calculation of Toxicity Using AutoDock

In this study, binding affinities between MUC-30 and targeted proteins that are overexpressed in breast cancer were proven by docking calculations. The estimated value of binding is presented from the best binding affinity energies (kcal·mol^−1^). MUC-30 was docked following the binding affinities with the targeted proteins EGFR, PgP, PR, NF-kB, ER*α*, and HER2. MUC-30 which is a semisynthetic compound to inhibit breast cancer with the targeted proteins' downloaded PDB IDs 2J6M, 6QEX, 4OAR, 1SVC, 3ERT, and 3WSQ showed scores as high as −8.7 kcal·mol^−1^, −5.0 kcal·mol^−1^, −6.0 kcal·mol^−1^, −7.1 kcal·mol^−1^, −8.6 kcal·mol^−1^, and −6.2 kcal·mol^−1^ performed by AutoDock, as shown in Table 4. Amongst the 6 targeted proteins, 2 targeted proteins that exhibited with energy values above tamoxifen binding for breast cancer receptors were EGFR and NF-kB. Therefore, breast cancer can be strongly inhibited by MUC-30 due to the inhibition of EGFR and NF-kB.

### 3.5. Cytotoxicity Study of MUC-30 and MUC-30-Loaded Polymeric Micelles

**Figure 4:**
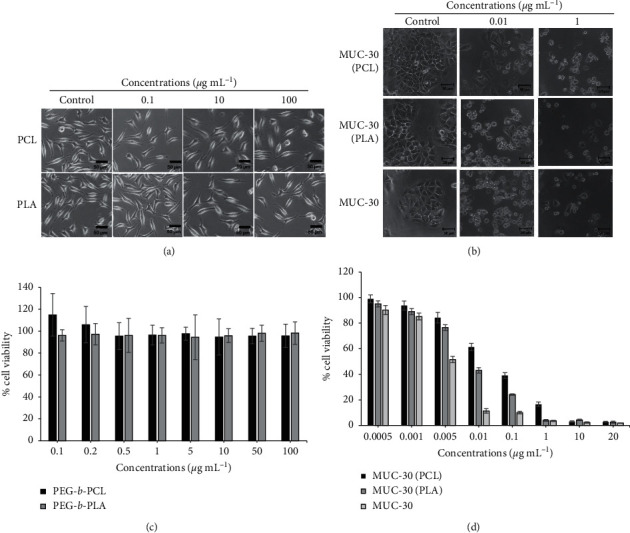
Cytotoxicity of MUC-30, MUC-30 loaded in PEG-b-PCL, and MUC-30 loaded in PEG-*b*-PLA against breast cancer cell line (MCF-7).

These block copolymers were tested to determine the nontoxicity level with normal cells. Fibroblast cells (L929) were treated with blank polymeric micelles, PEG-*b*-PLA and PEG-*b*-PCL micelles, and compared to a normal medium. Results demonstrate that these block copolymers are biocompatible, as shown in Figures 4(a) and 4(c). The cytotoxicity of MCF-7 after treatment with MUC-30 and MUC-30-loaded polymeric micelles was measured using an MTT assay and compared to the computational data. Results show that unencapsulated MUC-30 and encapsulated MUC-30 altered MCF-7 morphology as indicated by the circular shape as shown in Figure 4(b), which was a crucial sign of cell death; cytotoxicity directly relates increased concentration of MUC-30. This also corresponds to the changes in the morphology of MCF-7 (cell debris). Results show that the cell viability of unencapsulated MUC-30 from experiment (IC_50_ = 11 ± 0.39 ng·mL^−1^) compared to the computational calculation (IC_50_ of ER*α*: 0.49 *μ*M; PR: 42.54 *μ*M; EGFR: 0.40 *μ*M; HER2: 29.18 *μ*M; Pgp: 218.01 *μ*M; and NF-*κ*B: 6.69 *μ*M) was higher than MUC-30-encapsulated polymeric micelles presenting IC_50_ values of 37 ± 1.18 (PEG-*b*-PLA) and 75 ± 3.97 ng·mL^−1^ (PEG-*b*-PCL). Cytotoxicity of encapsulated MUC-30 was slightly lower than unencapsulated MUC-30. This was because of controlled release of MUC-30 from micelles.

**Figure 5:**
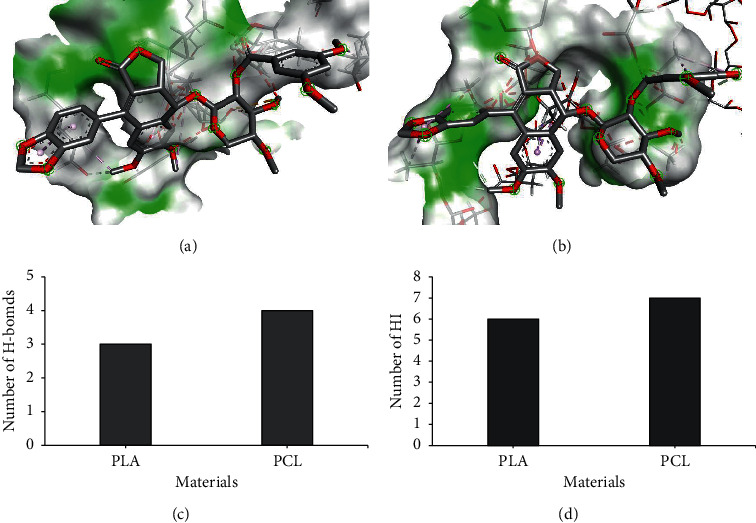
Typical snapshots from molecular docking by discovery studio showing (a) MUC-30 binding with the PCL surface and (b) MUC-30 binding with the PLA surface. Polymers are shown as surfaces, and MUC-30 is depicted using a line representation. The interaction of MUC-30 and polymers are shown as plotted bars in (c) hydrogen bonding and (d) hydrophobic interactions.

MUC-30 has a strong binding to PCL leading to the gradual release of MUC-30 from micelles compared to PLA which was not as strong as PCL. This leads to a faster release rate of MUC-30 from PLA compared to PCL which is consistent to the number of hydrogen bondings and hydrophobic interactions between MUC-30 and the polymer surface of PCL and PLA as shown in Figure 5, causing approximately two times more toxicity.

## 4. Conclusions

However, MUC-30 was somewhat more cytotoxic than MUC-30-loaded micelles, probably because MUC-30 can be transported into the nucleus of cells by the passive diffusion mechanism, while the drug-loaded micelles have to be internalized by endocytosis, release the loaded drugs, and then, diffuse through the endocytic before entering to nucleus of cells [32]. However, the properties of the drug-loaded nanoparticles were found to be safer with normal cells (L929) than those used with free drugs [33], which results in normal cell death as well. Although the effect of drug-loaded micelles suppressing cancer cells is less than that of a free drug, it was found that the inhibitory effectiveness of MUC-30-loaded micelles was still rather close to that of MUC-30 and did not affect normal cells [33]. For achieving minimum effective dose (MEC) in vivo, it was found that the drug-loaded micelles were more effective than the free drug. This is due to reduced drug clearance from the body [34] and efficiency in targeting cancer cells better through the EPR process [35].

This study has improved the MUC-30 properties and described the properties of encapsulated MUC-30 by computer simulation and in vitro experiment. Water solubility of MUC-30 was improved by encapsulation inside the micelle core and still inhibited MCF-7 growth. The release rate of MUC-30 through the micelle surface of PCL (*k* = 2.753; 4 HB; 7 HI) was slower than that of PLA (*k* = 7.236; 3 HB; 6 HI). In the cytotoxicity test, free MUC-30 (11 ng·mL^−1^) displayed higher MCF-7-inhibiting ability than MUC-30-loaded micelles. This was probably because MUC-30 was prolonged and gradually released from polymeric micelles following the Korsmeyer–Peppas model. The higher MUC-30 loading in PEG-*b*-PCL micelles inversely provided smaller particle size than PEG-*b*-PLA micelles which was most likely due to the strong interaction between MUC-30 and PCL. Finally, we believe understanding how MUC-30 interacts with a polymer and inhibits specific proteins will help the development of the hydrophobic natural compounds.

## Figures and Tables

**Table 1:** Micelle size, drug loading content (% DLC), encapsulation efficiency (% EE), and yields (% yields) of MUC30-loaded PEG-*b*-PCL and PEG-*b*-PLA micelles.

Block copolymer	Initial ratio of MUC-30 : polymer	Micelle size (nm)	DLC (%)	EE (%)	Yield (%)
PEG (5k)-*b*-PCL (5k)	1 : 9	39.76 ± 8.50	10.1 ± 0.1	70.6 ± 4.4	69.9 ± 4.0
	2 : 8	45.61 ± 3.34	19.1 ± 0.8	53.0 ± 1.7	54.0 ± 0.1
	3 : 7	55.68 ± 2.36	29.6 ± 0.3	64.8 ± 5.3	65.7 ± 5.0

PEG (5k)-*b*-PLA (5k)	1 : 9	52.67 ± 3.74	9.7 ± 0.3	55.6 ± 0.3	58.2 ± 1.8
	2 : 8	80.77 ± 2.77	18.9 ± 0.2	53.3 ± 1.0	56.5 ± 0.6
	3 : 7	93.1 ± 3.5	27.3 ± 0.7	9.4 ± 0.9	10.3 ± 0.8

**Table 2:** Mathematical equations for the models used to describe release characteristics of MUC-30 from PEG-*b*-PCL micelles.

Model	Equation	Parameter	*R*_abjusted_^2^
Zero order	*Q*_*R*_ = *k*_0_*t*	*k*_0_ = 266	−0.5096
First order	*Q*_*R*_=100(1 − *e*_1_^−*kt*^)	*k*_1_ = 0.003	−0.4505
Higuchi	*Q*_*R*_ = *K*_*H*_*t*^1/2^	*k*_*H*_ = 1.432	0.7238
Korsmeyer–Peppas	*Q*_*R*_ = *k*_KP_*t*^*n*^	*k*_KP_ = 2.753	0.9916
		*n* = 0.275	
Hixson–Crowell	*Q*_*R*_=100[1 − (1 − *k*_HC_*t*)^3^]	*k*_HC_ = 0.001	−0.4701
Hopfenberg	*Q*_*R*_=100[1 − (1 − *k*_HB_*t*)^*n*^]	*k*_HB_ = 0	−0.5273
		*n* = 136.29	
Baker–Lonsdale	1.5[1 − (1 − (*Q*_*R*_/100))^2/3^] − (*Q*_*R*_/100)=*k*_BL_t	*k*_BL_ = 0.00	0.7357
Quadratic	*Q*_*R*_=100(*k*_1_*t*^2^+*k*_2_*t*)	*k*_1_ = 0.000	0.5084
		*k*_2_ = 0.006	

**Table 3:** Mathematical equations for the models used to describe release characteristics of MUC30 from PEG-*b*-PLA micelles.

Model	Equation	Parameter	*R*_abjusted_^2^
Zero order	*Q*_*R*_ = *k*_0_*t*	*k*_0_ = 0.762	0.8912
First order	*Q*_*R*_=100(1 − *e*_1_^−*kt*^)	*k*_1_ = 0.009	−0.0665
Higuchi	*Q*_*R*_ = *K*_*H*_*t*^1/2^	*k*_*H*_ = 4.058	0.8128
Korsmeyer–Peppas	*Q*_*R*_ = *k*_KP_*t*^*n*^	*k*_KP_ = 7.236	0.9939
		*n* = 0.301	
Hixson–Crowell	*Q*_*R*_=100[1 − (1 − *k*_HC_*t*)^3^]	*k*_HC_ = 0.003	−0.1179
Hopfenberg	*Q*_*R*_=100[1 − (1 − *k*_HB_*t*)^*n*^]	*k*_HB_ = 0	−0.1229
		*n* = 617.721	
Baker–Lonsdale	1.5[1 − (1 − (*Q*_*R*_/100))^2/3^] − (*Q*_*R*_/100)=*k*_BL_t	*k*_BL_ = 0.000	0.8395
Quadratic	*Q*_*R*_=100(*k*_1_*t*^2^+*k*_2_*t*)	*k*_1_ = 0.000	0.5808
		*k*_2_ = 0.018	

**Table 4:** Binding energy of MUC-30 and tamoxifen with targeted proteins from AutoDock 4.

Ligands	Targeted proteins	PDB IDs	Binding affinity (kcal/mol)	IC_50_ for MUC-30 (*μ*M)
MUC-30	ER*α*	3ERT	−8.6	0.49
Tamoxifen	ER*α*	3ERT	−10.5	0.021
MUC-30	PR	4OAR	−6.0	42.54
Tamoxifen	PR	4OAR	−7.3	4.28
MUC-30	EGFR	2J6M	−8.7	0.40
Tamoxifen	EGFR	2J6M	−7.8	2.04
MUC-30	PgP	6QEX	−5.0	218.01
Tamoxifen	PgP	6QEX	−5.6	80.79
MUC-30	NF-kB	1SVC	−7.1	6.69
Tamoxifen	NF-kB	1SVC	−5.3	125.32
MUC-30	HER2	3WSQ	−6.2	29.18
Tamoxifen	HER2	3WSQ	−8.2	0.95

## Data Availability

The data used to support the findings of this study are available from the corresponding author upon request.
